# Dopamine-mediated action discovery promotes optimal behavior ‘for free’

**DOI:** 10.1186/1471-2202-12-S1-P138

**Published:** 2011-07-18

**Authors:** Ashvin Shah, Kevin Gurney

**Affiliations:** 1 Department of Psychology, University of Sheffield, Sheffield, S10 2TN, UK

## 

Redgrave and Gurney [1] have recently proposed a theory of action discovery in which the occurrence of an unexpected salient sensory event causes short-latency phasic dopamine (DA) neuron activity. Due to convergent projections from DA neurons and cortical neurons onto the striatum, the DA signal reinforces (i.e., biases the animal to repeat) preceding behaviour. In the case that the animal has caused the sensory outcome, continued repetition, along with variations in behaviour, allows the animal to discover the novel action-outcome contingency. A key aspect of this theory is that the DA signal decreases as the action-outcome association becomes predictable. In contrast is the well-known theory that the DA signal specifically represents a reward prediction error [2], and that the tendency to select behaviour is based on its ‘value’ (often represented as the expected subsequent cumulative costs and rewards). The latter theory is attractive in part because it conforms with Reinforcement Learning (RL), a computational formulation of learning from the consequences of actions [3]. RL describes well behaviour observed during many types of goal-directed tasks and allows behaviour to be described as optimal given a particular reward/cost function (e.g., cost per movement).

However, is a notion of optimality necessary to produce all behaviour thought to derive from DA-mediated mechanisms? If so, a representation of every movement’s cost and value is required to govern learning. The theory of action discovery [1] suggests that some behaviour is developed through much simpler mechanisms: the occurrence of the salient event rather than a prediction of some quantity (value) associated with each action selected. Here, we implement such a scheme in a learning agent that chooses discrete actions within a discrete state environment (analogous to pushing buttons). Only when the agent has reached a particular state (the salient event) is a reinforcement signal delivered. The tendency to select every action that was selected en route to that state is increased, but with a learning rate that decreases with temporal distance from the reinforcement signal (i.e., an eligibility trace). Importantly, no notion of optimality is represented.

**Figure 1:**
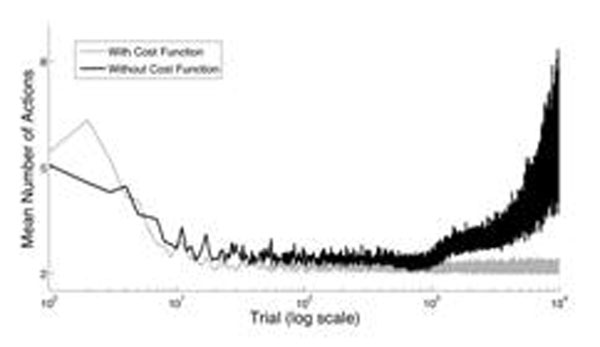


Figure 1 below illustrates the development of behavior due to our scheme (black line) and an RL scheme based on cost (grey). In both, the number of actions chosen en route to the salient event decreases toward a minimum. Such behavior is usually taken as evidence that it approaches optimal given some cost function on which learning depends. However, with our scheme, such behavior emerges ‘for free’ from the statistics of behavior over repeated trials. Actions that lie along the most direct path simply get reinforced at a greater rate than other actions.

If, as suggested by [1], learning is truncated after the outcome becomes predictable (via gradual extinction of phasic DA neuron activity), behavior under both schemes will stabilize to the most direct path. However, our scheme also predicts that, without such a truncation, prolonged ‘overlearning’ will result in suboptimal behavior where there is an increasing tendency to select spurious actions (see figure 1). Such behavior may be analogous to behavior arising from certain types of DA dysfunction. We discuss the implications of this learning scheme within the context of action discovery and describe additional predicted behaviors.
